# Effect of Fenugreek on vasomotor symptoms in menopausal women: A protocol for systematic review and meta-analysis

**DOI:** 10.1097/MD.0000000000020526

**Published:** 2020-06-05

**Authors:** Tingchao Wu, Rensong Yue, Mingmin He, Chenyi Xu

**Affiliations:** Hospital of Chengdu University of Traditional Chinese Medicine. Chengdu, PR China.

**Keywords:** Fenugreek, menopausal women, meta-analysis, protocol, vasomotor symptoms

## Abstract

**Background:**

Vasomotor symptoms (hot flashes or night sweats) are closely related to the impaired quality of life in menopausal women. Fenugreek is the ripe seed of *Trigonella foenum graecum* Linn. In China, this plant is used to relieve menopausal symptoms in women. Although recent studies have shown that fenugreek may have a good effect on the menopausal symptoms, there is no meta-analysis to systematically evaluate its efficacy in improving menopausal vasomotor symptoms.

**Methods:**

Randomized controlled trials that met the inclusion criteria will be retrieved in 5 English online databases and 4 Chinese online databases. The primary outcomes are changes in frequency and intensity of vasomotor symptoms that measured by validated scales. The secondary outcomes will include quality of life, blood hormone parameters, blood biochemical parameters, and adverse events. Heterogeneity of data will be assessed by *I*^2^ and Cochrane *Q* statistics. Sensitivity analysis and subgroup analysis will be performed to explore the sources of heterogeneity. Egger test and Begg test will be used to assess the publication bias. Finally, we will evaluate the quality of evidence by the GRADE approach. All the data statistics will be performed using the STATA 15.0 software.

**Results:**

All the results of will be published in a peer-reviewed journal.

**Conclusions:**

This meta-analysis will systematically evaluate the efficacy and safety of fenugreek in the treatment of menopausal vasomotor symptoms.

**OSF registration number:**

10.17605/OSF.IO/3BCY8.

## 1 Introduction

The term “menopause” was first created by C. P. L. de Gardanne in 1821, and is defined as the permanent amenorrhea in women for twelve consecutive months due to ovarian failure.^[1]^ Menopause usually occurs around the age of 51 years, mostly between 40 and 60 worldwide.^[2]^ Specifically, menopause is not a single point in time, but a dynamic period that generally lasts for several years, which is considered as an important transitional phase in a woman's life. According to the data from World Health Organization, 1.2 billion women will enter menopause by the year 2030.^[3]^

Due to the dramatic changes in hormone levels, about 4 out of 5 menopausal women experience physical or psychological symptoms, such as vasomotor symptoms, genitourinary symptoms, sleep disorder, cognitive decline, anxiety, and depression.^[4]^ Vasomotor symptoms are the most common menopausal symptoms, which experienced by approximately 80% of women in this transitional period.^[5]^ Vasomotor symptoms include hot flashes and night sweats, and hot flashes that occur during sleep are referred as night sweats. Hot flash is a sudden spontaneous sensation of heat in the upper body usually followed by flushing, sweating, chills, palpitations, and anxiety.^[6]^ These symptoms typically last from 1 to 5 minutes, occasionally up to 30 minutes.^[7]^ The frequency varies from several times a week to more than 10 times a day, and the average duration is 7.4 years.^[8]^ The results of a large multinational cross-sectional study^[9]^ suggest that vasomotor symptoms are closely related to the impaired quality of life in menopausal women. The pathophysiology of vasomotor symptoms is not yet clear but is generally thought to be associated with low estrogen levels.^[10]^

Hormone replacement therapy (oral estrogen or combined estrogen/progestogen) has been used for many years to relieve vasomotor symptoms in menopausal women. A meta-analysis of 24 trials showed that hormone therapy (HT) could significantly reduce the frequency and severity of hot flashes in menopausal women compared to placebo.^[11]^ However, an increased risk of venous thrombosis, coronary artery disease, stroke, breast cancer, and gallbladder disease following HT has been reported by the Women's Health Initiative.^[7]^

Fenugreek, the ripe seed of *Trigonella foenum graecum* Linn (family Fabaceae), is widely used as a condiment and traditional herbal medicine worldwide.^[12]^ Fenugreek contains many chemical ingredients such as alkaloids, saponins, flavonoids, coumarins, vitamins, and amino acids.^[13]^ Studies in animals and humans have reported numerous pharmacological effects of fenugreek, including anti-diabetes, lipid-lowering, anti-inflammation, antioxidation, antitumor, immunoregulation, and hepatoprotection, and so on.^[14]^ In China, this plant is also used by traditional Chinese medicine practitioners to relieve menopausal symptoms in women. Although some studies^[15–17]^ have reported the beneficial effects of fenugreek in menopausal women, its efficacy on vasomotor symptoms is still controversial. Accordingly, we will collect clinic evidence of fenugreek in the management of menopausal vasomotor symptoms, and conduct a meta-analysis to evaluate its efficacy and safety.

## 2 Methods

### 2.1 Search registration

This protocol has been registered on the Open Science Framework (OSF) platform, the registration number is 10.17605/OSF.IO/3BCY8. The reporting flow was conducted according to the Preferred Reporting Items for Systematic Reviews and Meta-analysis Protocols 2015 statement.^[18]^

### 2.2 Inclusion and exclusion criteria

#### 2.2.1 Study design

Only randomized controlled trials (RCTs) will be included in our study. Non-RCTs, observational studies, real-world studies, reviews, case reports, and animal experiments will be excluded.

#### 2.2.2 Participants

Women who were experiencing vasomotor symptoms due to menopausal, perimenopausal or postmenopausal period will be included. Women with vasomotor symptoms due to breast cancer treatment or surgical menopause will be excluded.

#### 2.2.3 Interventions

The intervention of the test group was fenugreek or fenugreek extract, and the control group was placebo-controlled. Moreover, studies using fenugreek or its extract as part of the compound prescription will not be included.

#### 2.2.4 Outcomes

The primary outcomes are changes in frequency and intensity of vasomotor symptoms (hot flashes or night sweats). The vasomotor symptoms must be measured by the Cooperman's index, Kupperman Index, Greene Climacteric scale, Menopause Rating Scale, or any other criteria for measuring menopausal symptoms. The secondary outcomes will include quality of life (based on generic validated scales), blood hormone parameters, blood biochemical parameters, and adverse events.

### 2.3 Study search

Two authors (Tingchao Wu and Mingmin He) will independently search the online databases (including PubMed, Embase, Web of Science, Cochrane Library, ClinicalTrials.gov, China National Knowledge Internet, VIP Information Chinese Periodical Service Platform, Wanfang Data Knowledge Service Platform, and Chinese Biomedicine Literature Database) for identifying the relevant studies. The search strategies will be performed using the Medical Subject Headings (MeSH) terms and free-text words. There will be no restrictions on the language and time of publication. The specific search strategy for PubMed database was shown in Table 1.

**Table 1:**
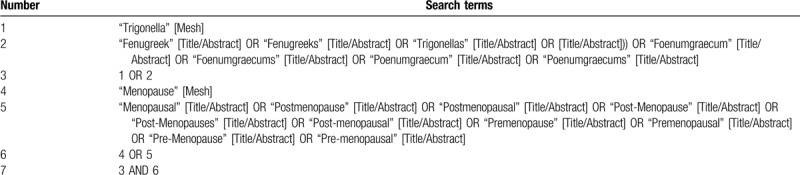
Search strategy for PubMed.

### 2.4 Study selection

**Figure 1:**
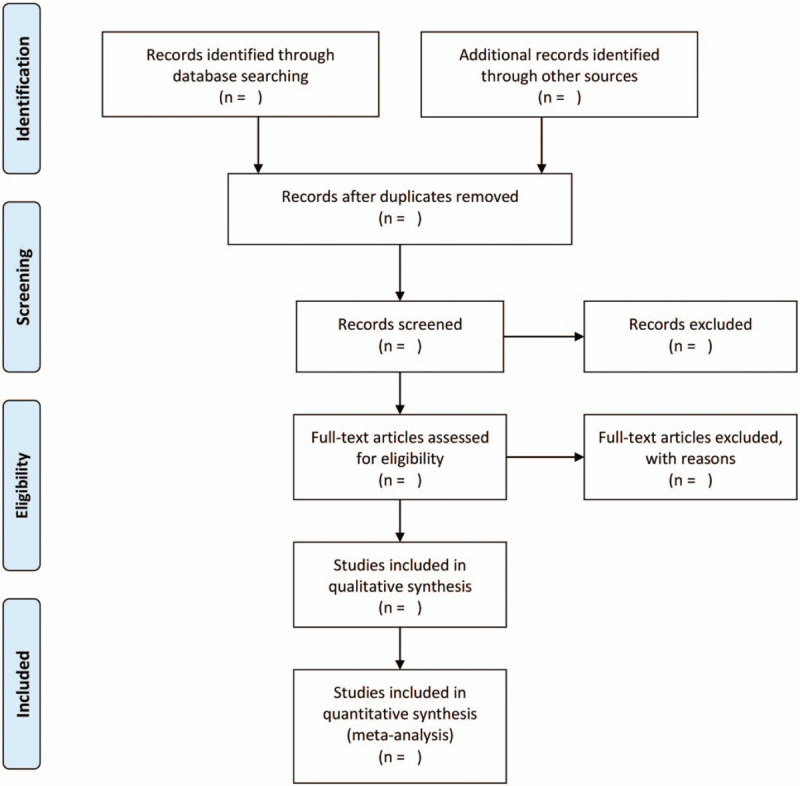
Flow chart of study selection.

EndNote X9 software^[19]^ will be used to manage the retrieved literature. Two independent authors (Tingchao Wu and Mingmin He) will screen the retrieved literature according to the inclusion and exclusion criteria. In the first step, the duplicated literature will be removed, and then the titles and abstracts will be reviewed. Subsequently, the full-text of the relevant literature will be investigated to assess their suitability for meta-analysis. Any discrepancies will be resolved by discussion with the corresponding author (Rensong Yue). We will show the detailed selection process by a flowchart (Fig.1).

### 2.5 Data extraction

We will extract relevant data from the included articles based on the following items: first Author; year of publication; country; study design; sample size (test/control group); age (test/control group); intervention (dosage form, dosage and duration of trial); outcomes in the test and control groups; Safety parameter and adverse event. If the data we need are not available in an article, we will try to obtain it by contacting the original investigators.

### 2.6 Risk of bias assessment

Two investigators (Tingchao Wu and Mingmin He) will independently assess the quality of the included studies according to the Cochrane Collaboration risk of bias tool.^[20]^ The content of the evaluation will cover 6 domains of bias: selection bias (“random sequence generation” and “allocation concealment”), performance bias (“blind of participants and personnel”), detection bias (“blinding of outcome assessment”), attrition bias (“incomplete outcome data”), reporting bias (“selective reporting”), and other bias. Any discrepancies will be judged by consultation with the corresponding author.

### 2.7 Data analysis

The STATA 15.0 software will be used for data statistics.^[21]^ For continuous data, mean difference between groups will be calculated if outcomes were reported on the same scale, while standardized mean difference will be calculated if outcomes were reported on different scales. Heterogeneity of the included studies will be assessed by *I*^2^ and Cochrane *Q* statistics. *I*^2^ < 50% with *P* > .05 indicate that there is no significant heterogeneity, the fixed-effect model will be used to combine effect sizes; otherwise, the random-effect model will be applied. The overall effects with *P*-value less than .05 will be considered statistically significant. If quantitative synthesis is not appropriate due to substantial heterogeneity, the results will be presented in table form.

### 2.8 Subgroup analysis

To better explore the sources of heterogeneity, subgroup analyses will be performed based on the following moderator variables: geographical area of subjects, assessment scale, dosage of fenugreek, dosage form of fenugreek, and intervention duration.

### 2.9 Sensitivity analysis

Sensitivity analysis will be used to evaluate the stability of the overall results by removing each trial sequentially. Then we will focus on the comparison of the pooled effect size and heterogeneity before and after sensitivity analysis.

### 2.10 Publication bias assessment

If more than 10 trials are finally included in our study, Egger's linear regression test^[22]^ and Begg test^[23]^ will be used to assess the potential publication bias. *P*-value less than .05 will be considered as statistically significant.

### 2.11 Grading the quality of evidence

The GRADE approach will be used to evaluate the quality of evidence for the entire study.^[24]^ The evaluation includes 5 domains: risk of bias (study design and execution), imprecision, indirectness, inconsistency, and publication bias. Each domain will be defined as “very low”, “low”, “moderate” or “high”.

### 2.12 Ethics and dissemination

Because meta-analysis is a secondary analysis of the published studies, ethical approval is not applicable. All the results of this meta-analysis will be published in a peer-reviewed journal.

## 3 Discussion

Due to the potential adverse effects of hormone replacement therapy, many menopausal women are trying to seek alternatives to the treatment.^[25–27]^ Natural and safe botanicals, especially those that can be used as food ingredients, are a popular new option for managing menopausal discomforts. Several systematic reviews have been conducted to evaluate the efficacy of some botanicals on menopausal symptoms, such as black cohosh, red clover, ginseng, and hypericum perforatum.^[28–31]^ Fenugreek is a relatively new botanical in the field of scientific research, and recent studies have shown that it may have a good effect on the menopausal symptoms. However, there is no meta-analysis to summarize the relevant clinical trials and systematically assess its efficacy in improving menopausal vasomotor symptoms.

To improve the overall quality of this meta-analysis, only RCTs will be included in the analysis. Since women with breast cancer may develop prematurely menopausal symptoms as a result of chemotherapy or estrogen-blocking drugs,^[32]^ we will exclude these patients. Moreover, in order to reduce the heterogeneity across trials and evaluate the effect of fenugreek more accurately, we will also exclude women with menopausal symptoms due to ovarian surgery. In China, herbal compounds are more commonly used in clinical practice than single herbs. Therefore, to avoid the influence of trials using 2 or more herbs on the overall analysis results, studies using fenugreek as part of the compound prescription will not be included. Since vasomotor symptoms are difficult to measure objectively, to ensure the quality of this study, we will only include those studies that use validated scales for outcome assessment. Sreeja S et al^[33]^ provided evidence for the estrogenic activities of fenugreek and believed that it has the potential to be an alternative for HT. To assess whether the effects of fenugreek on menopausal women are related to the phytoestrogenic effects, we will also include blood hormone parameters as a secondary outcome. Blood biochemical parameters and adverse events will be used as evidence for safety assessment.

This study has some limitations. First, our search is limited to Chinese and English databases, which may lead to the omission of some valuable RCTs. Second, significant heterogeneity may exist across the included studies due to different assessment scales. If data permitting, we will explore the sources of heterogeneity through subgroup analysis.

This meta-analysis is the first study to systematically evaluate the efficacy of fenugreek in the treatment of menopausal vasomotor symptoms, which will provide additional evidence for the use of herbal supplements during menopause.

## Author contributions

**Conceptualization:** Tingchao Wu, Rensong Yue.

**Data curation:** Tingchao Wu, Mingmin He.

**Formal analysis:** Tingchao Wu, Chenyi Xu.

**Investigation:** Tingchao Wu, Mingmin He.

**Methodology:** Tingchao Wu.

**Project administration:** Rensong Yue.

**Software:** Tingchao Wu, Chenyi Xu.

**Visualization:** Tingchao Wu.

**Writing – original draft:** Tingchao Wu.

**Writing – review and editing:** Tingchao Wu, Mingmin He.
